# Summer undergraduate biomedical research program for underrepresented minority students in a rural, low-income state

**DOI:** 10.3389/fpubh.2024.1395942

**Published:** 2024-05-23

**Authors:** Michael E. Anders, Latrina Y. Prince, Tremaine B. Williams, Robert E. McGehee, Billy R. Thomas, Antino R. Allen

**Affiliations:** ^1^Academic Affairs, University of Arkansas for Medical Sciences, Little Rock, AR, United States; ^2^Graduate School, University of Arkansas for Medical Sciences, Little Rock, AR, United States; ^3^Biomedical Informatics, College of Medicine, University of Arkansas for Medical Sciences, Little Rock, AR, United States; ^4^Department of Pediatrics, College of Medicine, University of Arkansas for Medical Sciences, Little Rock, AR, United States; ^5^Department of Pharmaceutical Sciences, College of Pharmacy, University of Arkansas for Medical Sciences, Little Rock, AR, United States

**Keywords:** minority groups, female, education, medical, undergraduate, biomedical research, mentors, surveys and questionnaires

## Abstract

**Introduction:**

Diversity can enhance the agenda and quality of biomedical research, but a dearth of underrepresented minorities and women serve as biomedical researchers. The study purpose was to examine the impact of the a summer undergraduate research program on self-efficacy in research, scientific communication, and leadership as well as scientific identity, valuing objectives of the scientific community, and intent to pursue a biomedical research career.

**Methods:**

Underrepresented minority and female undergraduate students participated in a mentored research experience in a rural, low-income state.

**Results:**

Students' self-efficacy in research, scientific communication, and leadership as well as scientific identity, valuing objectives of the scientific community, and intent to pursue a biomedical research career increased post-program compared to pre-program.

**Conclusion:**

This study supports implementation of a biomedical summer undergraduate research program for URM and women in a poor, rural, settings.

## Introduction

Diversity in biomedical research can reduce health disparities (1). A diverse biomedical research workforce promotes a more comprehensive research agenda, including research on diseases with a disproportionate impact on underrepresented minorities (URM), primarily because researchers from populations with the greatest burden of disease tend to identify and apply solutions (2). Diversity can also enhance research quality. Ethnically or gender diverse research teams publish manuscripts in journals with higher impact factors and receive more citations, and peer reviewers perceive them as higher quality (3, 4). Yet, in many cases, biomedical researchers lack resemblance to the population they serve, because of a lack of diversity.

Indeed, a dearth of URM and women serve as biomedical researchers. Though URM make up 34.6% of the U.S. population, URM hold <6% of appointments among biomedical research faculty. African Americans constitute <3% of researchers with NIH research grants (5–7). Similarly, women hold less than one-third of tenured biomedical research faculty positions (8). A large gap exists in the number of URM and female biomedical researchers compared to their white and male counterparts. Reaching population parity may be an ideal goal, but there are many barriers to achieving parity including persistent incidents of discrimination, bias, stereotyping, and exclusion of minority students. These barriers collectively maintain the gap and result in low numbers of URM and women who pursue and attain a degree in science, technology, engineering, mathematics, and medicine (STEMM) (9).

Degree attainment in STEMM by URM and women is not narrowing this gap. Though URM students pursue STEMM majors at nearly the same rate as White and Asian students, unfortunately, the number who persist and graduate is low (10). African American and Hispanic STEMM students graduate at a rate of 34% and 43%, compared to much higher rates in White students (58%) (11). Indeed, African Americans earned only 9% and Hispanics 15% of STEMM bachelor degrees (12). Moreover, women earned 85% of health related degrees but less than one-fourth of the degrees for other sciences (13). A small pool of URM and female students are in a position to pursue graduate degrees in STEMM careers.

Pipeline programs for undergraduate students promote academic achievement and URM entry into STEMM and healthcare fields (14–16). The Survey of Undergraduate Research Experiences indicated most students had a sustained or increased interest in post-graduate studies after a summer research experience and reported an increase in their independence, motivation to learn, and undergraduate course participation (14). Further, pipeline programs for undergraduate students have reported improvement in research skills and increased matriculation into graduate biomedical research programs (15, 16). Still, observed gains are minor compared to the needs of URM and women in the population. A significant need exists for more interventions to improve diversity and inclusion in STEMM education and careers. Summer Undergraduate Research Programs exist throughout the country, including the rural south (17). For example, the University of Mississippi Medical Center offers the Summer Undergraduate Research Experience, which has many attributes similar to those of our Summer undergraduate Research Program (SURP) (18). In contrast, our SURP explicitly seeks to enroll URM and female students. Further, a scarcity of reports exist in the literature that report effective strategies, interventions, and evaluation outcomes in STEMM undergraduate pipeline programs in rural, impoverished southern states such as Arkansas, which account for 20% of Americans and where URM have less access to STEMM careers and chronic health disparities have the largest impact (19).

Arkansas is a rural, low-income southern state. It is the sixth most rural state, with 41% of the population living in rural areas compared to 14% nationally (20). Arkansas has the sixth highest poverty rate, with 17% of the population living below the poverty line compared to 13% nationally (21). Moreover, 37% of the Arkansas population is low-income, and 27% receive Medicaid coverage (22). In rural low-income households, many parents have little formal education. Thus, many of these parents lack expectations for children to pursue a STEMM education and career (23).

At the University of Arkansas for Medical Sciences (UAMS), <7% of the faculty are minorities, which constrains efforts to engage URM communities using URM faculty for direct advocate-to-community engagement. In particular, it constrains biomedical research domains with a focus on cardiovascular and blood disorders, as heart disease is the leading cause of death in the United States, including individuals of most racial and ethnic groups (24, 25). African Americans in Arkansas with reduced cardiovascular function have a significantly higher number of hospitalizations and readmissions than White Americans (26, 27). Further, prevalence of cardiovascular-related chronic conditions, such as congestive heart failure, and mortality risk are nearly twice the national average in many Arkansas counties where poverty persists. Thus, Arkansas is a fitting environment for assessing innovative biomedical training programs to address the complex health needs of its communities. UAMS, the only academic health center in Arkansas, implemented a SURP to increase the volume and quality of URM researchers with interests in cardiovascular, pulmonary, or hematologic research in the biomedical workforce pipeline.

SURP is a nine-week research experience, with the overarching goal to increase the percentage of URM students who pursue STEMM graduate degrees and careers in research or health professions. Specific aims were to: (a) Recruit a diverse group of academically talented and enthusiastic undergraduate students interested in pursuing careers in cardiovascular, pulmonary, or hematologic research, (b) Support and cultivate successful and rewarding mentor-mentee relationships, (c) Develop and promote student leadership and communication skills, (d) Stimulate underrepresented, minority, and disadvantaged students' interest in research and health-related careers, and (e) Evaluate the program and its activities to ensure student satisfaction and program effectiveness.

The purpose of this study was to examine the impact of the SURP on the students' self-efficacy in research, scientific communication, and leadership as well as impact on the extent they identified with science, valued objectives of the scientific community, and their intent to pursue a biomedical research career. We believed other academic health science centers in rural, low-income states could use our findings to help develop or enhance similar mentored research experiences for underrepresented minority undergraduate students.

## Materials and methods

### Setting

The setting was at UAMS, an academic health science medical center in a rural, low-income southern state.

### Participants

The participants were URM, female, and first-generation undergraduate students enrolled in the 2022 and 2023 SURP.

### Study design

The study design was a one-group, retrospective pre-post, and qualitative framework analysis.

### Procedures

Each year, the SURP recruited and accepted up to 13 URM, female, or first-generation undergraduate students from colleges and universities throughout the U.S. The program has received an average of 70 applications per year. Program directors reviewed each application and selected participants based on several requirements. Participants were U.S. citizens who had completed at least 1 year of undergraduate studies with a minimum of 2.5 GPA. Applicants also answered questions about their research experience and career goals. In addition, applicants provided two letters of recommendation from faculty. Once accepted into the program, UAMS hired SURP participants as temporary student employees for 9-weeks during the summer.

Before the program began, based on the students' interest and background, SURP administrators paired each student with a faculty mentor, who supervised the students' summer research project and worked closely with the student throughout the summer. A SURP administrator had formal mentor training at the Center for the Improvement of Mentored Experiences in Research in Wisconsin and is currently participating in a campus wide effort to facilitate research mentor and mentee training at UAMS. The SURP program provided funding for research supplies. In mid-summer, students introduced their research project to other SURP participants and program staff. The research project culminated with each student delivering a research presentation in an open forum to all faculty mentors, program participants, and SURP administrators as well as invited faculty and family members.

In addition to the research project, students attended weekly research and professional development seminars. The seminars exposed students to various areas of research on the UAMS campus and sought to increase their knowledge about biomedical science and research careers. Students received a comprehensive tour of all of the research areas on the campus and had the opportunity to shadow health professionals who worked in the clinics and main hospital. At the conclusion of the program, students completed an evaluation. This manuscript presents the results of that evaluation.

### Theoretical framework

The Tripartite Integration Model of Social Influence served as the theoretical framework for the program and the evaluation (28, 29). This framework helps explain how people socially integrate into a community. It features three social variables that influence the extent someone integrates into a community: (a) self-efficacy, (b) identity, and (c) values. In the context of this study, scientific self-efficacy refers to the extent the students believed in their ability to meet the challenges of planning, conducting and communicating about scientific experiments. Scientific identity was the extent the students had a gratifying definition of themselves as scientists. Scientific community values reflected how aligned the students were with the objectives of the scientific community. Together, scientific self-efficacy, science identity, and scientific community values can influence biomedical research career intention. Effective mentorship, a key component of our SURP, can promote self-efficacy, identity, and values (30, 31).

### Measures

Demographic measures included undergraduate status, first-generation college student, gender, race, and ethnicity. We elicited the students' satisfaction with the program, including relevance, extent the SURP stimulated interest in a biomedical research career, quality, and recommending the SURP to other students.

The Mentoring Competency Assessment examines the competencies and skills of scientists who serve as research mentors (32). The instrument features a seven-point Likert scale (1 = Not at all skilled; 7 = Extremely skilled) to assess 26 skills needed for six competencies, including:: (a) Communication, (b) Alignment of expectations, (c) Assessment of understanding, (d) Fostering independence, (e) Addressing diversity, and (f) Promoting professional development. For example, skills for communication included active listening, providing constructive feedback, and developing a trusting relationship. Aligning expectations included setting goals and developing strategies to meet them. Assessing understanding included assessing the mentee's knowledge and ability. Fostering independence included motivating mentees, building their confidence, and stimulating creativity. Addressing diversity included accounting for different backgrounds. Promoting professional development included facilitating networking and setting career goals.

The investigators used a retrospective post then pre survey method for several measures. With this method, students made two separate sets of ratings, both post-SURP and pre-SURP, only after participating in the SURP. First, the students made post-SURP “NOW” or “AFTER” ratings about their knowledge, skills, attitude, or behaviors. Then, we asked the students to reflect back and rate the same knowledge, skills, attitude, or behaviors “BEFORE” the SURP. Previous research on survey methodology demonstrated that compared to conventional pre and post measures, the retrospective post then pre design reduced participants' overestimation of pre measures and, therefore, avoided underestimation of effect size (33, 34). Post then pre measures included self-efficacy in research competencies, scientific communication, and leadership as well as scientific identity, scientific values, and intention to pursue a research career in biomedical science (35–38). To compare post then pre outcomes for these constructs, the investigators totaled the score for each construct and divided each total by the number of possible points, to arrive at a score on a 100-point scale.

Additionally, students provided written reflections to qualitative inquiries about science identity and values as well as intention to pursue a biomedical research career. Seven open-ended questions assessed program strengths and weaknesses as well as how the program influenced career or academic choices.

### Analysis

Descriptive statistics summarized data for demographics, satisfaction, mentoring competencies as well as self-efficacy in research competencies, scientific communication, leadership scientific identity, and scientific values. Median (Mdn) with interquartile ranges (IQR) expressed central tendency and the spread of the middle half of the nonparametric data.

The Wilcoxon rank sum test for nonparametric variables compared pre- and post- self-efficacy for research competencies, scientific communication, leadership scientific identity, scientific values, and intention to pursue a research career in biomedical science. The equation (r) = Z/square root of N estimated effect size. Alpha was *p* < 0.05.

For written responses to qualitative inquiries, the investigators used a conceptual analysis, with top-down deductive themes and inductive sub-themes. MAXQDA (2021) software analyzed qualitative data.

### Ethics

The UAMS institutional review board determined this study was not human subject research.

## Results

### Participants

Twenty-two (*N* = 22) students participated, with 12 in 2022 and 10 in 2023. Ninety-five percent were URM, women, or first-generation college students (see Table 1).

**Table 1:** Characteristics of the students (*N* = 22).

**Characteristic**	***n* (%)**
**Undergraduate status**	
Sophomore	6 (27)
Junior	11 (50)
Senior	5 (23)
**First generation college student**	
Yes	9 (41)
No	13 (59)
**Gender**	
Male	7 (32)
Female	15 (68)
**Gender identity**	
Man	7 (32)
Woman	13 (62)
Non-binary	1 (5)
Preferred not to answer	1 (5)
**Hispanic, Latin, or Spanish**	
Yes	3 (14)
No	19 (86)
**Race**	
American Indian or Alaska native	1 (5)
Asian	4 (19)
Black or African American	3 (14)
Native Hawaiian or Pacific Islander	2 (10)
White	9 (43)
Other or unknown	2 (10)
Preferred not to answer	1 (5)

### Satisfaction

**Figure 1:**
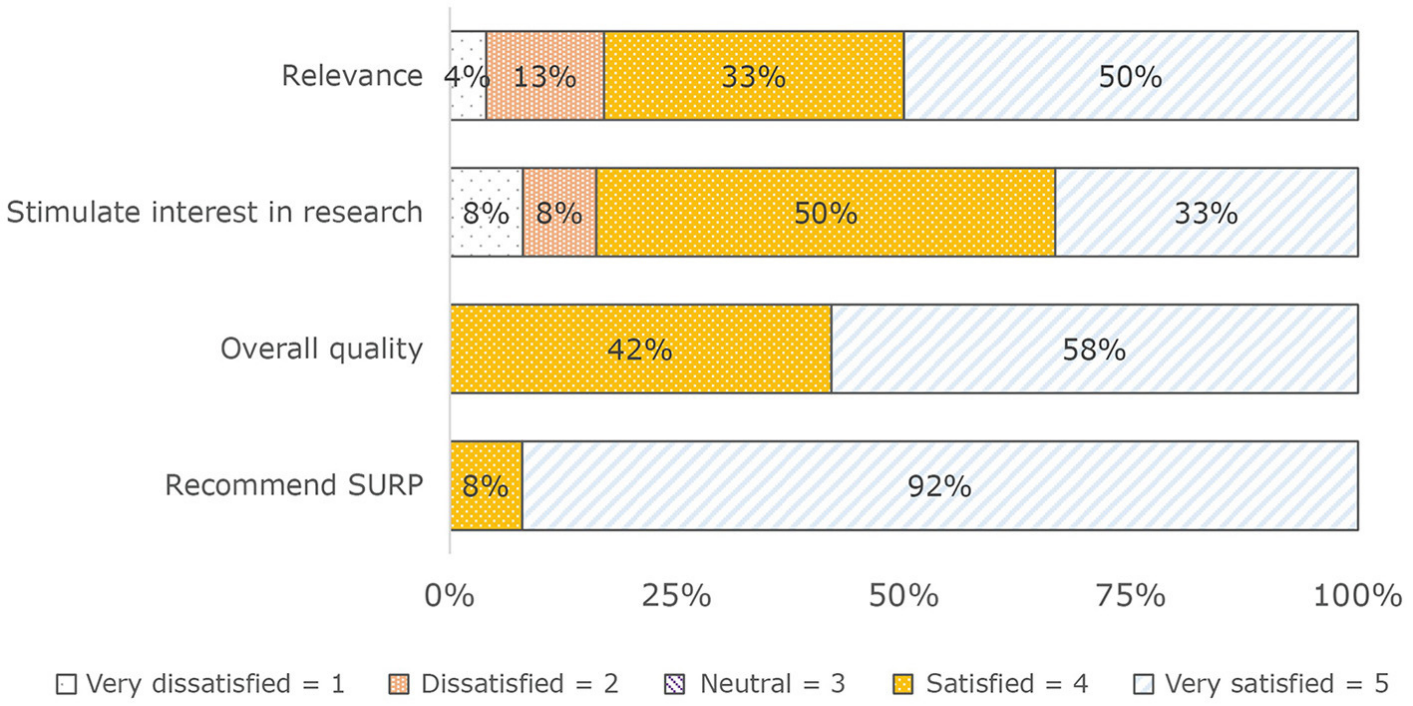
General satisfaction with the SURP (*N* = 22).

Students were satisfied with the SURP. They rated the SURP positively for: (a) Relevance (83%), (b) Extent the SURP stimulated interest in a biomedical research career (83%), (c) Quality (100%), and (d) Recommending the SURP to other students (100%) (see Figure 1). Similarly, with the exception of shadowing experiences in the clinical setting, most students were satisfied with each educational activity outside of their mentored research experience (Table 2).

**Table 2:** Satisfaction with specific SURP activities (*N* = 22).

**Activity**	**1**	**2**	**3**	**4**	**5**	**M (SD)**	**Mnd (IQR)**
SURP overview in orientation	0 (0)	0 (0)	2 (9)	6 (27)	14 (64)	4.6 (0.67)	5 (1)
Radiation and lab safety	0 (0)	0 (0	4 (18)	6 (27)	12 (55)	4.4 (0.79)	5 (1)
Preparing a curriculum vitae	0 (0)	0 (0)	3 (14)	4 (18)	15 (68)	4.6 (0.74)	5 (1)
Writing and talking science	0 (0)	0 (0)	4 (18)	7 (32)	11 (50)	4.3 (0.78)	4.5 (1)
Data acquisition, lab notebooks, management, and ownership	0 (0)	1 (5)	1 (5)	8 (36)	12 (55)	4.4 (0.8)	5 (1)
Careers panel discussion	0 (0)	1 (4)	0 (0)	7 (32)	14 (64)	4.6 (0.74)	5 (1)
Ethics case studies	0 (0)	0 (0)	1 (5)	6 (27)	15 (68)	4.6 (0.58)	5 (1)
Shadowing experience in clinical setting	3 (14)	2 (9)	2 (9)	2 (9)	13 (59)	3.9 (1.5)	5 (2)
Networking seminar	0 (0)	1 (5)	2 (9)	7 (32)	12 (55)	4.4 (0.85)	5 (1)
From discovery to clinical trials	0 (0)	1 (5)	3 (14)	7 (32)	11 (50)	4.3 (0.88)	4.5 (1)
Research presentations	0 (0)	0 (0)	4 (18)	4 (18)	14 (64)	4.5 (0.8)	5 (1)

SURP, Summer Undergraduate Research Program; M, mean; SD, standard deviation; Mnd, median; IQR, interquartile range. Scale: 1 = Poor; 5 = Excellent.

### Mentorship

For the mentored research experiences, the students' median rating for all domains of the mentee-mentor relationship was 7, on a seven-point scale (see Table 3).

**Table 3:** Mentoring (*N* = 22).

**Competency**	**1**	**2**	**3**	**4**	**5**	**6**	**7**	**M (SD)**	**Mnd (IQR)**
Active listening	0 (0)	0 (0)	1 (4)	2 (9)	1 (5)	4 (18)	14 (64)	6.3 (1.2)	7 (1)
Constructive feedback	0 (0)	0 (0)	1 (5)	1 (5)	0 (0)	4 (18)	16 (73)	6.5 (1.1)	7 (1)
Trust-based relationship	0 (0)	1 (5)	1 (5)	1 (5)	1 (5)	3 (14)	15 (68)	6.2 (1.1)	7 (1)
Accommodate different communication styles	0 (0)	0 (0)	1 (5)	3 (14)	2 (9)	2 (9)	14 (64)	6.2 (1.5)	7 (1)
Communication	0 (0)	0 (0)	1 (5)	4 (18)	0 (0)	1 (5)	16 (73)	6.1 (1.3)	7 (2)
Clear expectations	0 (0)	0 (0)	1 (5)	2 (9)	1 (5)	2 (9)	16 (73)	6.4 (1.2)	7 (1)
Align expectations	0 (0)	0 (0)	2 (9)	1 (5)	3 (14)	3 (14)	13 (59)	6.1 (1.3)	7 (2)
Consider professional & personal differences	0 (0)	0 (0)	0 (0)	2 (9)	2 (9)	2 (9)	16 (73)	6.5 (1)	7 (1)
Help set mentee's research goals	0 (0)	0 (0)	0 (0)	1 (5)	1 (5)	4 (18)	16 (73)	6.6 (0.8)	7 (1)
Develop strategies for mentee to meet goals	0 (0)	0 (0)	0 (0)	1 (5)	2 (9)	3 (14)	16 (73)	6.6 (0.86)	7 (1)
Estimate mentee's scientific knowledge	0 (0)	0 (0)	1 (5)	0 (0)	2 (9)	5 (23)	14 (64)	6.4 (1.1)	7 (1)
Estimate mentee's ability to conduct research	0 (0)	0 (0)	1 (5)	0 (0)	2 (9)	5 (23)	14 (64)	6.4 (1)	7 (1)
Enhance mentee's knowledge and abilities	0 (0)	0 (0)	1 (5)	0 (0)	1 (5)	6 (27)	14 (64)	6.5 (0.96)	7 (1)
Motivate mentee	1 (5)	0 (0)	0 (0)	2 (9)	1 (5)	2 (9)	16 (73)	6.3 (1.5)	7 (1)
Build mentee's confidence	1 (5)	0 (0)	0 (0)	2 (9)	1 (5)	4 (18)	14 (64)	6.2 (1.5)	7 (1)
Stimulate mentee's creativity	1 (5)	0 (0)	0 (0)	2 (9)	2 (9)	3 (14)	14 (64)	6.1 (1.5)	7 (1)

M, mean; SD, standard deviation; Mnd, median; IQR, interquartile range. Scale: 1 = Not at all skilled; 7 = Extremely skilled.

### Qualitative inquiry

When asked to discuss the most valuable aspect(s) of the SURP, major themes were networking, hands-on lab training and skills, mentee-mentor relationships, and research experience (Table 4). The participants' suggestions for improving the SURP were: (a) Better pairing of mentees with mentors, (b) Fewer and more interactive seminars, and (c) More clinical and shadowing opportunities. We then asked participants to discuss how the SURP affected their ability to conduct research. Many indicated they had a better understanding of research, learned new lab techniques, and had more interest in research. We also wanted to know if the SURP affected how participants identified with the role of scientists. An overwhelming number of participants indicated they had a “better understanding of research and the role of a scientist.” The next question asked participants to discuss how the SURP affected how they value the objectives of the scientific community. Several participants mentioned more value and appreciation for the scientific community, including a better understanding of the importance of science in relation to medicine as well as more appreciation and respect for the scientific community. How the SURP affected the intent to pursue a science related research career received mixed responses. Some participants indicated the SURP confirmed their intent to pursue a science related research career, while others said it prompted their intent to pursue both an MD/PhD, and a few indicated it stimulated their interest in conducting clinical research as a physician.

**Table 4:** Qualitative inquiry.

**Deductive theme**	**Inductive sub-themes**	**Illustrative quotes**
Most valuable aspects	•Networking •Hands-on lab skills •Hearing from professionals	The most valuable aspect of the program was my mentor. I also enjoyed the curriculum vitae seminar. I've actually (not) understood how to create one until this summer. Prior to the SURP I had no research experience, and here I got to learn and do real research for the first time.
Suggestions for Improvements	•More guidance and communication from administration •Better clinical opportunities •More interactive seminars	Provide more opportunities for students to shadow (in clinical settings) (Spend) less time on seminars and more time in lab
Biomedical research	•Enhanced ability to conduct research •Enhanced identification with role of scientists •Heightened intention to pursue career scientific research	I can identify a topic, acquire data, assess the evidence, develop conclusions, and share the knowledge I learn thanks to SURP. I believe I exhibit all of the qualities of a scientist. Being able to work directly with (scientists) made me appreciate what they do. It made me see the value in doing research for medicine. It made me affirm my joy for science and understand I can pursue a career in something I'm passionate about. Originally, I was just trying to pursue my MD, but now, since attending SURP, I want to pursue the MD/PhD.

### Self-efficacy in research competencies

**Figure 2:**
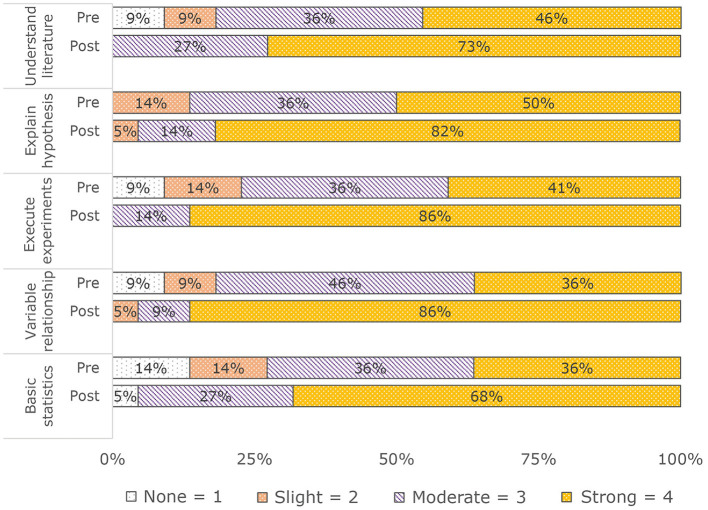
Pre/post research competencies (*N* = 22).

Post-SURP, students reported higher self-efficacy score for each research competency: (a) Understand literature Pre: M (SD) = 3.2 (0.9); Mnd (IQR) = 3 (1); Post: M (SD) = 3.7 (0.5); Mnd (IQR) = 4 (1); (b) Explain hypothesis (Pre: M (SD) = 3.4 (0.7); Mnd (IQR) = 3.5 (1); Post: M (SD) = 3.8 (0.5); Mnd (IQR) = 4 (0); (c) Execute experiments: Pre: M (SD) = 3.1 (1); Mnd (IQR) = 3 (1); Post: M (SD) = 3.9 (0.4); Mnd (IQR) = 4 (0); (d) Variable relationship: Pre: M (SD) = 3.1 (0.9); Mnd (IQR) = 3 (1); Post: M (SD) = 3.8 (0.5); Mnd (IQR) = 4 (0); (e) Basic statistics: Pre: M (SD) = 3 (1); Mnd (IQR) = 3 (2); Post: M (SD) = 3.6 (0.7); Mnd (IQR) = 4 (1) (see Figure 2).

### Self-efficacy in scientific communication and leadership

Post-SURP, students reported higher self-efficacy scores for each facet of scientific communication we measured as well as measures for internal and external leadership (see Tables 5, 6).

**Table 5:** Pre/post scientific communication (*N* = 22).

**Communication skill**	**Strongly disagree**	**Disagree**	**Neutral**	**Agree**	**Strongly agree**	**M (SD)**	**Mnd (IQR)**
**Write using correct grammar**							
Pre	0 (0)	1 (5)	4 (18)	9 (41)	8 (36)	4.1 (0.9)	4 (1)
Post	0 (0)	0 (0)	0 (0)	5 (23)	17 (77)	4.8 (0.4)	5 (0)
**Write abstract**							
Pre	1 (5)	6 (27)	3 (14)	3 (14)	9 (41)	3.6 (1.4)	4 (3)
Post	0 (0)	1 (5)	2 (9)	5 (23)	14 (64)	4.5 (0.9)	5 (1)
**Manage anxiety about writing**							
Pre	0 (0)	2 (9)	5 (23)	6 (27)	9 (41)	4.0 (0.1)	4 (2)
Post	0 (0)	0 (0)	0 (0)	7 (32)	15 (68)	4.7 (0.5)	5 (1)
**Excel in scientific presentations**							
Pre	0 (0)	5 (23)	5 (23)	4 (18)	8 (36)	3.7 (1.2)	4 (2)
Post	0 (0)	0 (0)	3 (14)	4 (18)	15 (68)	4.6 (0.8)	5 (1)
**Give an oral presentation**							
Pre	0 (0)	0 (0)	8 (36)	5 (23)	9 (41)	4.1 (0.9)	4 (2)
Post	0 (0)	0 (0)	2 (9)	5 (22)	16 (70)	4.6 (0.7)	5 (1)
**Require little assistance with speaking and presenting**							
Pre	1 (5)	2 (9)	7 (32)	5 (23)	7 (32)	3.7 (1)	4 (2)
Post	0 (0)	0 (0)	2 (9)	4 (18)	16 (73)	4.6 (0.7)	5 (1)
**Defend point of view in a scientific discussion**							
Pre	0 (0)	1 (5)	9 (41)	4 (18)	8 (36)	3.9 (1)	4 (2)
Post	0 (0)	0 (0)	2 (9)	5 (23)	15 (68)	4.6 (0.7)	5 (1)
**Answer questions in scientific discussion**							
Pre	0 (0)	2 (9)	7 (32)	5 (23)	8 (36)	3.9 (1)	4 (2)
Post	0 (0)	0 (0)	1 (5)	7 (32)	14 (64)	4.6 (0.6)	5 (1)
**Speak using correct grammar**							
Pre	0 (0)	0 (0)	2 (9)	9 (41)	11 (50)	4.4 (0.7)	4.5 (1)
Post	0 (0)	0 (0)	0 (0)	6 (27)	16 (73)	4.7 (0.5)	5 (1)
**Manage worries about speaking**							
Pre	0 (0)	0 (0)	4 (18)	6 (27)	12 (55)	4.4 (0.8)	5 (1)
Post	0 (0)	0 (0)	0 (0)	6 (27)	16 (73)	4.7 (0.5)	5 (1)
**Ask question or add comment during discussion in the lab**							
Pre	0 (0)	2 (9)	5 (23)	6 (27)	9 (41)	4 (1)	4 (2)
Post	0 (0)	0 (0)	1 (5)	5 (23)	16 (73)	4.7 (0.6)	5 (1)
**Ask a question in front of audience after a presentation**							
Pre	0 (0)	4 (18)	5 (23)	3 (14)	10 (46)	3.9 (1.2)	4 (2)
Post	0 (0)	1 (4)	2 (9)	5 (22)	14 (64)	4.5 (0.9)	5 (1)
**Use scientific speaking style**							
Pre	0 (0)	3 (14)	5 (23)	5 (23)	9 (41)	3.9 (1.1)	4 (2)
Post	0 (0)	0 (0)	1 (5)	6 (27)	15 (68)	4.6 (0.6)	5 (1)
**Introduce self and research**							
Pre	0 (0)	2 (9)	5 (23)	4 (18)	11 (50)	4.1 (1.1)	4.5 (2)
Post	0 (0)	0 (0)	0 (0)	5 (23)	17 (77)	4.8 (0.4)	5 (0)

M, mean; SD, standard deviation; Mnd, median; IQR, interquartile range.

**Table 6:** Pre/post leadership (*N* = 22).

	**1**	**2**	**3**	**4**	**5**	**6**	**7**	**M (SD)**	**Mnd (IQR)**
**Self-awareness and confidence**									
**I can identify my strengths and weaknesses**									
Pre	0 (0)	0 (0)	1 (5)	1 (5)	4 (18)	10 (45)	6 (27)	5.9 (1)	6 (2)
Post	0 (0)	0 (0)	0 (0)	0 (0)	0 (0)	8 (36)	14 (64)	6.6 (0.49)	7 (1)
**I am confident in my ability to get things done**									
Pre	0 (0)	1 (5)	0 (0)	3 (14)	1 (5)	7 (32)	10 (46)	6.0 (1.4)	6 (1)
Post	0 (0)	0 (0)	0 (0)	0 (0)	2 (9)	4 (18)	16 (73)	6.6 (0.66)	7 (1)
**I always know how to get the best out of situations**									
Pre	0 (0)	1 (5)	0 (0)	4 (18)	2 (9)	6 (27)	9 (41)	5.8 (1.4)	6 (2)
Post	0 (0)	0 (0)	0 (0)	0 (0)	4 (18)	5 (23)	13 (59)	6.4 (0.8)	7 (1)
**I can help group members to reach the group's target**									
Pre	0 (0)	0 (0)	1 (5)	4 (18)	0 (0)	8 (36)	9 (41)	5.9 (1.3)	6 (2)
Post	0 (0)	0 (0)	0 (0)	0 (0)	3 (14)	5 (23)	14 (64)	6.5 (0.74)	7 (1)
**I am able to affirm my beliefs and values**									
Pre	0 (0)	0 (0)	1 (5)	3 (14)	3 (14)	4 (18)	11 (50)	6.0 (1.3)	6.5 (2)
Post	0 (0)	0 (0)	0 (0)	0 (0)	1 (5)	5 (23)	16 (73)	6.7 (0.57)	7 (1)
**Interpersonal relations**									
**I can establish very good relationships with the people I work with**									
Pre	0 (0)	0 (0)	0 (0)	0 (0)	5 (23)	6 (27)	11 (50)	6.3 (0.83)	6.5 (1)
Post	0 (0)	0 (0)	0 (0)	0 (0)	0 (0)	7 (32)	15 (68)	6.7 (0.48)	7 (1)
**I am sure I can communicate with others, going right to the heart of the matter**									
Pre	0 (0)	0 (0)	0 (0)	1 (5)	5 (23)	5 (23)	11 (50)	6.2 (0.96)	6.5 (2)
Post	0 (0)	0 (0)	0 (0)	0 (0)	1 (5)	6 (27)	15 (68)	6.6 (0.58)	7 (1)
**I can successfully manage relationships with all members of a group**									
Pre	0 (0)	0 (0)	0 (0)	1 (5)	5 (23)	5 (23)	11 (50)	6.2 (0.96)	6.5 (2)
Post	0 (0)	0 (0)	0 (0)	0 (0)	0 (0)	6 (27)	16 (73)	6.7 (0.46)	7 (1)

M, mean; SD, standard deviation; Mnd, median; IQR, interquartile range. Scale: 1 = Absolutely false; 7 = Absolutely true.

### Science identity

**Figure 3:**
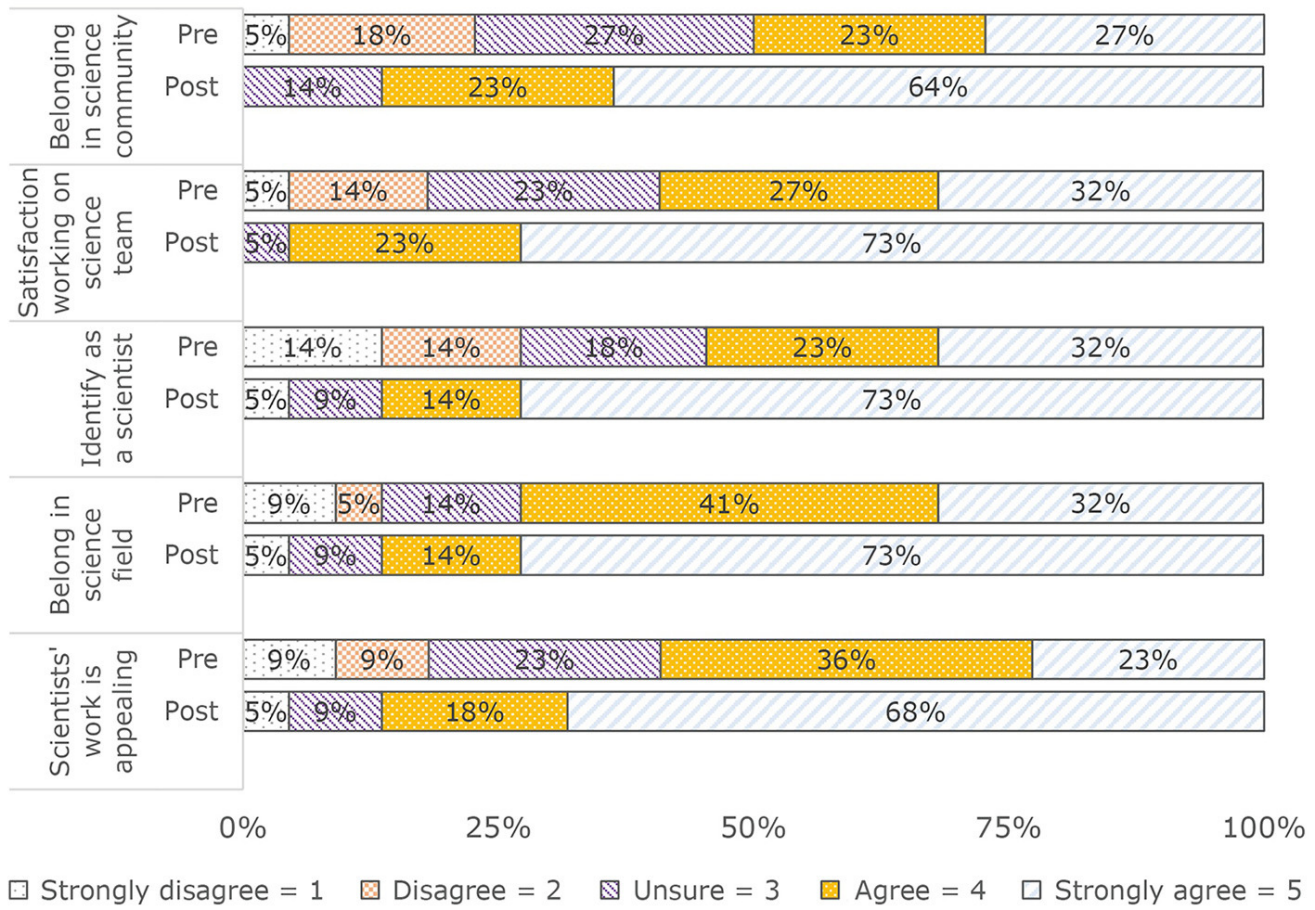
Pre/post identify with science (*N* = 22).

Post-SURP, students reported higher scores for each facet of science identity: (a) Belonging in science community: Pre: M (SD) = 3.5 (1.2); Mnd (IQR) = 3.5 (2); Post: M (SD) = 4.5 (0.7); Mnd (IQR) = 5 (1); (b) Satisfaction working on science team: Pre: M (SD) = 3.2 (1.2); Mnd (IQR) = 4 (2); Post: M (SD) = 4.7 (0.6); Mnd (IQR) = 5 (1); (c) Identify as a scientist: Pre: M (SD) = 3.5 (1.4); Mnd (IQR) = 4 (3); Post: M (SD) = 4.7 (0.6); Mnd (IQR) = 5 (1); (d) Belonging in science field: Pre: M (SD) = 3.8 (1.2); Mnd (IQR) = 4 (2); Post: M (SD) = 4.5 (0.9); Mnd (IQR) = 5 (1); (e) Scientists' work is appealing: Pre: M (SD) = 3.6 (1.2); Mnd (IQR) =4 (1); Post: M (SD) = 4.5 (1); Mnd (IQR) = 5 (1) (see Figure 3).

### Valuing objectives of the scientific community

**Figure 4:**
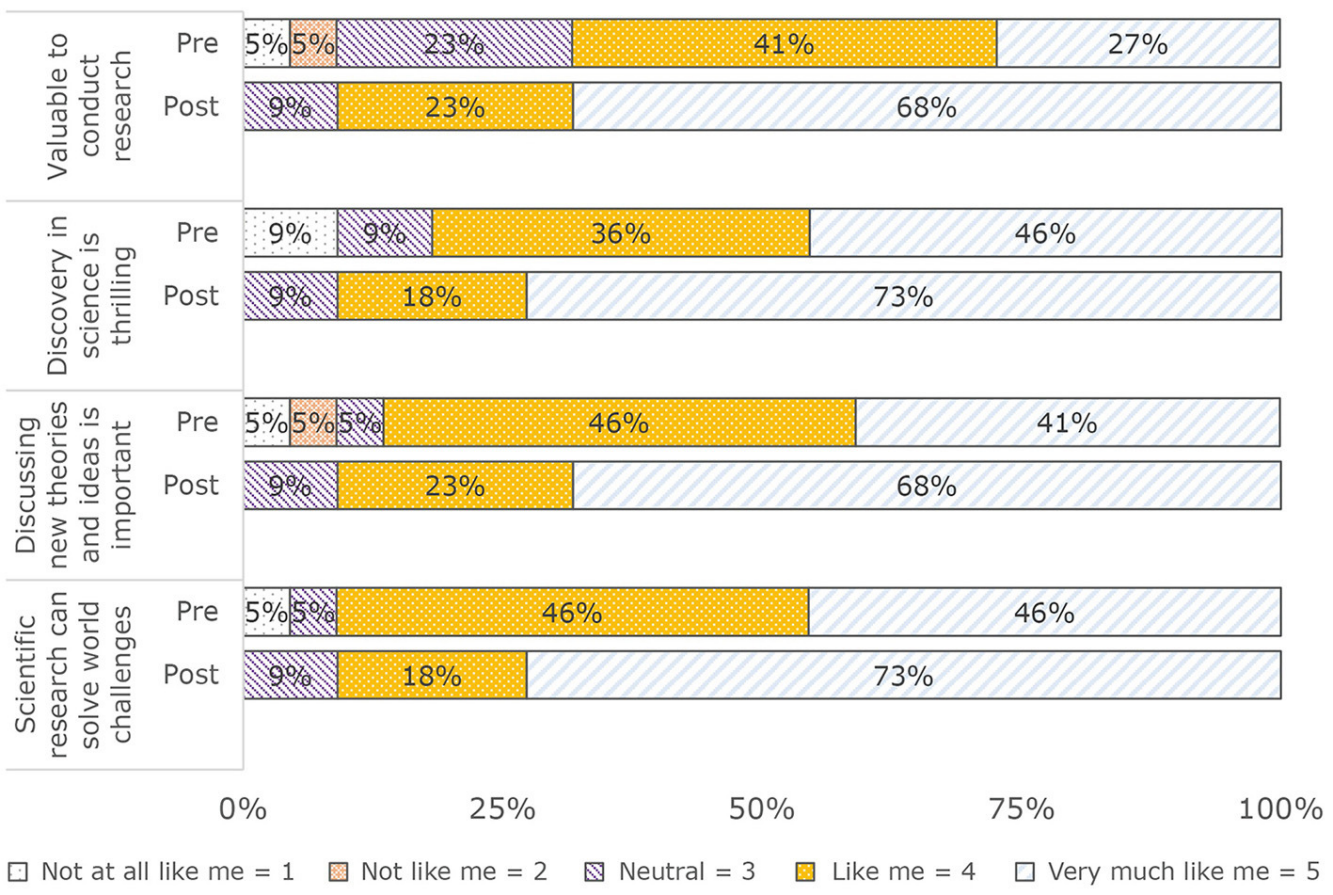
Pre/post value scientific community objectives (*N* = 22).

Post-SURP, students reported higher scores for each facet of valuing the objectives of the scientific community: (a) Valuable to conduct research: Pre: M (SD) = 3.8 (1); Mnd (IQR) = 4 (2); Post: M (SD) = 4.6 (0.7); Mnd (IQR) = 5 (1); (b) Discovery in science is thrilling: Pre: M (SD) = 4.2 (1); Mnd (IQR) = 4 (1); Post: M (SD) = 4.6 (0.7); Mnd (IQR) = 5 (1); (c) Discussing new theories and ideas is important: Pre: M (SD) = 4.1 (1); Mnd (IQR) = 4 (1); Post: M (SD) = 4.6 (0.7); Mnd (IQR) = 5 (1); (d) Scientific research can solve world challenges: Pre: M (SD) = 4.3 (0.9); Mnd (IQR) = 4 (1); Post: M (SD) = 4.6 (0.7); Mnd (IQR) = 5 (1) (see Figure 4).

### Comparison of pre- and post-outcomes for construct totals and intention to pursue biomedical research career

Compared to pre-SURP measures, post-SURP measures were significantly higher with medium or large effect sizes (see Table 7).

**Table 7:** Pre/post comparisons of construct totals (*N* = 22).

**Variable**	**M (SD)**	**Mnd (IQR)**	**Ranks^a^**	**Effect size^b^**	***p-*value^c^**
**Research competencies** ^d^			Positive 11	0.64	0.003
Pre	0.78 (0.2)	0.78 (0.4)	Negative 1		
Post	0.94 (0.1)	1.0 (0.1)	Ties 10		
**Communication** ^d^			Positive 15	0.73	<0.001
Pre	0.79 (0.2)	0.80 (0.4)	Negative 0		
Post	0.93 (0.1)	1.0 (0.2)	Ties 7		
**Leadership: self-awareness and confidence** ^d^			Positive 15		
Pre	0.84(0.2)	0.87 (0.2)	Negative 1	0.63	0.003
Post	0.94 (0.1)	0.97 (0.1)	Ties 6		
**Leadership: interpersonal relations** ^d^			Positive 9		
Pre	0.89 (0.1)	0.93 (0.2)	Negative 0	0.57	0.007
Post	0.95 (0.1)	1.0 (0.1)	Ties 13		
**Identify with science** ^d^			Positive 16		
Pre	0.72 (0.2)	0.76 (0.4)	Negative 1	0.73	<0.001
Post	0.91 (0.2)	1.0 (0.2)	Ties 5		
**Value scientific community objectives** ^d^			Positive 14		
Pre	0.82 (0.2)	0.80 (0.2)	Negative 0	0.71	<0.001
Post	0.93 (0.1)	1.0 (0.2)	Ties 8		
**Intention to pursue a science related research career** ^d^			Positive 15		
Pre	0.71 (0.3)	0.75 (0.4)	Negative 4	0.43	0.03
Post	0.80 (0.3)	0.93 (0.4)	Ties 3		

M, mean; SD, standard deviation; Mnd, median; IQR, interquartile range. ^a^Ranks compare each student's pre and post scores. ^b^Effect size (r) = Z/square root of N; r 0.10–0.30 = small effect size; r between 0.30 and 0.5 = medium effect size; r > 0.50 = large effect size. ^c^Wilcoxon Rank Sum Test. ^d^Total score of all construct items/Total possible highest score of all construct items.

## Discussion

### Summary of the results

Study results demonstrated students were satisfied with the SURP and rated their mentee-mentor relationships highly. Compared to pre-SURP, self-efficacy in research, scientific communication, and leadership as well as increased science identity and valuing the objectives of the scientific community improved post-SURP. Further, compared to pre-SURP, post-SURP students indicated a stronger intention to pursue a career in biomedical research. Qualitative inquiry revealed the SURP encouraged students' to strongly identify with science, value the scientific community's objectives, and intend to pursue a biomedical research career.

### Explanation of the results

The Tripartite Integration Model of Social Influence helps explain our findings (28, 29). The model provides a framework for explaining how a URM student orients to a scientific community/research environments (11). The model posits individuals socialize into a group through three processes: building scientific efficacy (i.e., following rules and norms for rewards, minimizing punishment), building scientific identity (i.e., developing a social identity that includes the environment), and internalizing scientific community values (i.e., embracing and sharing the values of the group/research (31)). Our results suggest: (a) The SURP contributed to the students' increased self-efficacy and (b) Increased self-efficacy generated by the SURP program may have influenced the students'intention to pursue a scientific career. Immersive mentored research projects and hands-on laboratory training help explain students' increased self-efficacy in research, scientific communication, and leadership. In turn, increased self-efficacy likely explains the students' increased science identity and valuing objectives of the scientific community, which may explain their enhanced intention to pursue a biomedical research career.

### Comparison with other studies

Our results were consistent with that of other studies that demonstrated the influence of summer research programs on intention to pursue a career in the biomedical research workforce. A previous report about our SURP demonstrated students had positive and sustained interactions with mentors and the SURP influenced their career goals (39). The current study extends these results. Hernandez et al. (31) found development of science identity and values was associated with intention to pursue a STEMM career in undergraduates in several summer research programs. A qualitative study of the Arkansas IDeA Network of Biomedical Research Excellence Summer Research Fellowship found providing mentored research experiences for undergraduate students enhanced their interest in pursuing a career in the biomedical research workforce (40). A study in Idaho revealed improvements in science identity were associated with transitioning from summer undergraduate research to graduate school. The study found 91.4% of summer research students entered a scientific or healthcare related career, while 71% pursued graduate training, citing improved awareness of science as a cause of pursuing in the workforce pipeline (41). Similarly, the K-INBRE, a summer undergraduate research program in Kansas, identified career awareness as a critical component of program success, with 37% of students matriculating into graduate programs, 19% into medical school, and 12% into health-related professions (42). Yassa et al. (43) reported improvement in science self-efficacy and identity as well as visualization of a career as a scientist, in students predominantly from a Historically Black Universities after they participated in a mentored summer research program at the University of California. Both the Short-term Research Education Program to Increase Diversity in Health-Related Research Fellowships and the Integrative Organismal Systems Physiology Fellowships enrolled underrepresented students (44). However, none of the programs in these studies specifically targeted URM and female students in a rural, low-income southern state and reported outcomes that included science identity and alignment of values with the scientific community, which are components of the Tripartite Integration Model of Social Influence.

Summer research programs increased the probability of retaining students in the pipeline by expanding career awareness components of the programs beyond their common elements, such as lectures and lab exposure. This expansion could include a framework for career intention, such as the Tripartite Integration Model of Social Influence. Effective career awareness programs do the following: (a) Engage family, teachers, and friends of the students and (b) Use socialization initiatives to rebrand science, such as mitigating negative gender connotations (45). However, unlike the SURP, previous summer research programs do not universally integrate these strategies for enhancing career intention.

### Limitations

This study had several limitations. Prominent among them was the use of self-reported data. Students could have provided inaccurate responses. However, the questions were anonymous, evidence exists for validity of the outcomes measures, and our findings were consistent with that of other studies. Additionally, the retrospective post then pre design could have introduced a recall bias. However, we used this design to help avoid response-shift bias seen with traditional pre-post measures, that is, students' pre-SURP self-evaluation overestimation of their self-efficacy, values, and intentions, which could result in underestimation of the SURP outcomes (31, 32).

### Implications

Our study supports implementation of a biomedical research pipeline for URM and women in a poor, rural, and settings. Future studies should examine long-term academic and career outcomes and, ultimately, the impact on health disparities.

## Data availability statement

The raw data supporting the conclusions of this article will be made available by the authors, without undue reservation.

## Author contributions

MA: Conceptualization, Data curation, Formal analysis, Investigation, Methodology, Writing – original draft, Writing – review & editing. LP: Data curation, Formal analysis, Funding acquisition, Project administration, Writing – original draft, Writing – review & editing. TW: Data curation, Funding acquisition, Writing – original draft, Writing – review & editing. RM: Funding acquisition, Project administration, Supervision, Writing – original draft, Writing – review & editing. BT: Funding acquisition, Project administration, Resources, Supervision, Writing – original draft, Writing – review & editing. AA: Funding acquisition, Project administration, Supervision, Writing – original draft, Writing – review & editing.
